# Deep Temporal Clustering of Pathological Gait Patterns in Post-Stroke Patients Using Joint Angle Trajectories: A Cross-Sectional Study

**DOI:** 10.3390/bioengineering12010055

**Published:** 2025-01-11

**Authors:** Gyeongmin Kim, Hyungtai Kim, Yun-Hee Kim, Seung-Jong Kim, Mun-Taek Choi

**Affiliations:** 1 Department of Intelligent Robotics, Sungkyunkwan University, Suwon 16419, Republic of Korea; hn04008@skku.edu; 2 School of Mechanical Engineering, Sungkyunkwan University, Suwon 16419, Republic of Korea; gudxo1229@skku.edu; 3 Department of Physical and Rehabilitation Medicine, Sungkyunkwan University School of Medicine, Suwon 16419, Republic of Korea; yunkim@skku.edu; 4 Myongji Choonhey Rehabilitation Hospital, Seoul 07378, Republic of Korea; 5 Department of Biomedical Engineering, College of Medicine, Korea University, Seoul 02841, Republic of Korea

**Keywords:** post-stroke, hemiplegia, gait patterns, kinematic data, time-series data, deep clustering

## Abstract

Rehabilitation of gait function in post-stroke hemiplegic patients is critical for improving mobility and quality of life, requiring a comprehensive understanding of individual gait patterns. Previous studies on gait analysis using unsupervised clustering often involve manual feature extraction, which introduces limitations such as low accuracy, low consistency, and potential bias due to human intervention. This cross-sectional study aimed to identify and cluster gait patterns using an end-to-end deep learning approach that autonomously extracts features from joint angle trajectories for a gait cycle, minimizing human intervention. A total of 74 sub-acute post-stroke hemiplegic patients with lower limb impairments were included in the analysis. The dataset comprised 219 sagittal plane joint angle and angular velocity trajectories from the hip, knee, and ankle joints during gait cycles. Deep temporal clustering was employed to cluster them in an end-to-end manner by simultaneously optimizing feature extraction and clustering, with hyperparameter tuning tailored for kinematic gait cycle data. Through this method, six optimal clusters were selected with a silhouette score of 0.2831, which is a relatively higher value compared to other clustering algorithms. To clarify the characteristics of the selected groups, in-depth statistics of spatiotemporal, kinematic, and clinical features are presented in the results. The results demonstrate the effectiveness of end-to-end deep learning-based clustering, yielding significant performance improvements without the need for manual feature extraction. While this study primarily utilizes sagittal plane data, future analysis incorporating coronal and transverse planes as well as muscle activity and gait symmetry could provide a more comprehensive understanding of gait patterns.

## 1. Introduction

As the number of strokes increases, the treatment of motor disorders in the lower limbs, which are one of its after-effects, is also gaining attention [1]. The majority of post-stroke hemiplegic patients have difficulty walking to perform daily activities, even if most survivors eventually recover their motor ability [2]. Effective rehabilitation of post-stroke patients requires personalized care that takes into account different patterns of gait impairment [3].

To identify the gait patterns that show the successive motion from stance to swing during the time interval, machine learning (ML), which efficiently handles multi-dimensional data, is used in gait analysis [4]. Begg and Kamruzzaman [5] explored the potential of support vector machines (SVMs) in automatically recognizing changes in gait patterns due to ageing. Nutakki et al. [6] reported that the extracted gait features from an accelerometer could be classified by various ML-based algorithms and used to analyze motor dysfunction. To interpret the gait patterns of post-stroke patients, Mulroy et al. [7] analyzed temporal and kinematic parameters from post-stroke patients’ walking via non-hierarchical clustering and classified them into four groups. Similarly, Sánchez et al. [3] used unsupervised sparse K-means clustering to analyze spatiotemporal features and forces in patients. Also, Kim et al. [8] classified different motor impairments based on extracted kinematic features from time-series gait cycles of post-stroke patients by harnessing the simultaneous clustering and classification (SCC) method [9], which alleviates the limitation of conventional clustering.

Despite their achievements, conventional ML approaches face limitations due to manual feature extraction [10]. This process to extract features by users is subject to human bias, which may lead to overlooking relevant features or including irrelevant ones and impact the model’s accuracy [11]. Additionally, it is labor-intensive, requiring significant time and resources to manually interpret the features from big data [12]. To overcome these constraints, deep learning (DL) offers a promising solution by reducing the need for manual feature engineering, as it can automatically learn hierarchical representations directly from raw data [13].

Recent advances in DL have significantly contributed to the field of gait analysis and clinical diagnostics such as stroke rehabilitation. Rojek et al. [14] demonstrated the efficacy of artificial neural networks (ANNs) and fuzzy logic in analyzing post-stroke gait data, proposing an AI-based system that automates gait classification and facilitates low-cost, rapid assessment in clinical settings. Konz et al. [15] introduced ST-DeepGait, a spatiotemporal deep learning model that analyzes patterns in the movements of multiple human joints to enable gait recognition. Their model employs a recurrent neural network (RNN) architecture, leveraging spatiotemporal graphs that are pre-defined by users. Additionally, Wang et al. [2] developed a deep neural network (DNN) utilizing inertial measurement units (IMUs) to detect and classify stroke gait abnormalities, distinguishing between different pathological gait patterns.

However, while DL has shown promise in reducing reliance on manual feature extraction, many studies still depend on pre-processed kinematic data or pre-defined spatiotemporal features to analyze the patterns. For instance, Konz et al. [15] employed spatiotemporal graphs that were manually defined to enhance the accuracy of their DL model, and Rojek et al. [14] used an ANN with pre-processed gait features. These approaches demonstrate the potential of DL but also reveal that fully automated, end-to-end clustering directly from raw joint-level gait cycles remains an area for further exploration. Moreover, in real-world clinical settings, most acquired datasets lack labels, and even when labels are present, they are often manually assigned by humans, introducing potential biases [16]. To ensure a more realistic and unbiased analysis, it is crucial to apply unsupervised learning methods that utilize unlabeled data, allowing the model to identify and cluster gait patterns based solely on their intrinsic characteristics.

The objective of this cross-sectional study aims to implement an end-to-end DL approach that directly utilizes time-series gait cycle data as model input, eliminating the need for manual feature extraction. An optimized set of hyperparameters is identified for unsupervised gait pattern clustering of post-stroke hemiplegic patients. Furthermore, to confirm the significance of the optimized clusters, statistics on various gait characteristics for each cluster are presented as a supporting result. To our knowledge, this is the first study to apply deep learning clustering to identify similar gait patterns directly using joint-level gait cycle data from hemiplegic post-stroke patients in an end-to-end manner.

The rest of this paper is organized as follows: Section 2 describes the dataset, preprocessing method, and DL-based framework used for analysis. Section 3 presents the process of hyperparameter optimization and the clustering outcomes, including the characteristics of selected gait groups. Section 4 discusses the implications of the results, comparing them to previous studies and highlighting the potential clinical applications. Finally, Section 5 concludes the paper by summarizing key contributions.

## 2. Materials and Methods

### 2.1. Data Collection

To develop a deep learning model, this study used the data from our previous study [8]. The data, collected at Samsung Medical Center (SMC) in South Korea from May 2017 to August 2022, mainly consisted of time-normalized joint angle trajectories, motion-captured during independent gaits of post-stroke hemiplegic patients. In addition, the spatiotemporal, kinematic, and clinical assessment data were collected. All measurements were performed with the consent of each patient and approved by the SMC Institutional Review Board (SMC 2017-11-081). There were 74 sub-acute hemiplegic post-stroke patients aged from 23 to 86. Among the patients, 42 were male and 32 were female. In addition, 51 patients had a diagnosis of ischemic strokes and 23 of hemorrhagic strokes. The average age, height, and weight with a standard deviation of total patients were 59.66 (±15.26) years, 164.13 (±8.69) cm, and 65.67 (±10.80) kg, respectively.

Using eight cameras at a 60 Hz sample rate via the Motion Analysis Corporation’s motion capture system, marker trajectory data were collected during walking along a ten-meter track. The guideline of the Helen-Hayes marker set [17] was applied to the patient’s lower limb to ensure consistent placement and accurate tracking of anatomical landmarks. Raw marker trajectories, representing the 3D positions of reflective markers, were processed using Orthotrak software 6.6.4 [18] to compute joint angles and velocities [19]. This process involved applying inverse kinematics to the marker data, allowing for the estimation of hip, knee, and ankle joint angles throughout the gait cycle [20]. The software utilizes predefined anatomical models and calibrations to accurately derive joint kinematics from the spatial displacement of markers [21]. Considering that gait recovery rates gradually decrease over time [22], the raw kinematic data and gait characteristics were collected at 2, 3, 4, 6, 8, 10, 12, and 24 weeks after onset for each patient.

### 2.2. Preprocess

As input data for the deep learning, we used joint angles and angular velocity trajectories in the sagittal plane measured at specific weeks after onset as an instance. Kinematic data were not measured for all patients over the eight week points after onset due to inability to walk independently, early discharge, or refusal to participate. As a result, 219 instances were formed by excluding non-existent data and collecting measured ones regardless of patients and weeks. Table 1 summarizes the number of instances of kinematic data after onset.

In deep learning, a tensor is a tool for representing high-dimensional data within machine learning and deep learning frameworks [23]. The joint angle trajectories were converted into a tensor, which is directly used in the DL model, minimizing the interference by human in end-to-end gait pattern analysis. For this study, the tensor had a dimension of 219 instances and 600 features representing the concatenated joint angle and angular velocity trajectories from the hip, knee, and ankle.

### 2.3. Deep Temporal Clustering for Gait Pattern

**Figure 1:**
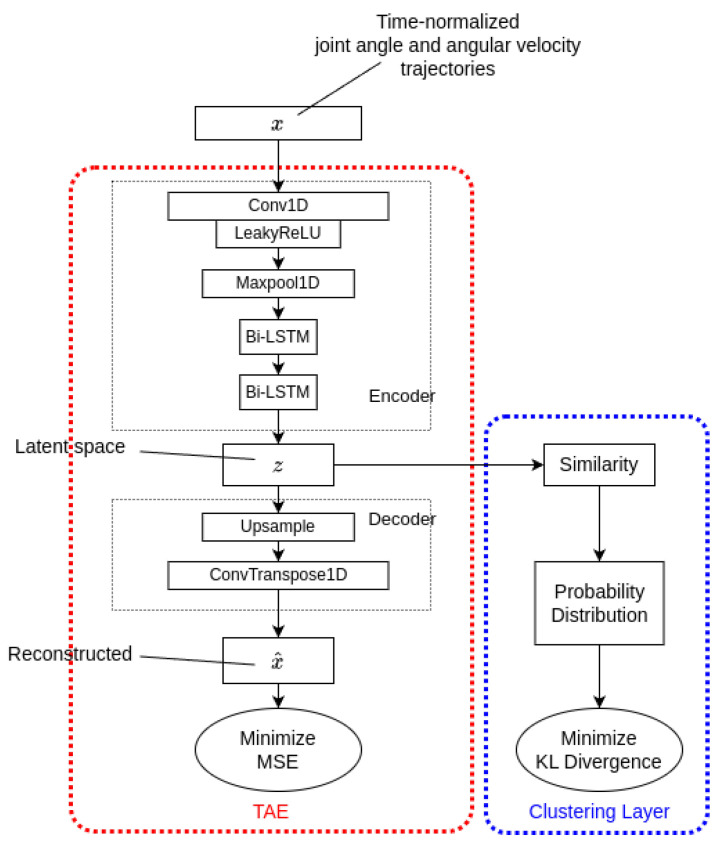
The architecture of deep temporal clustering for gait patterns.

For gait pattern analysis, we introduce Deep Temporal Clustering for Gait Pattern (DTCGP), which is based on the existing Deep Temporal Clustering algorithm (DTC) by Madiraju et al. DTC is used for unsupervised learning of time-series data in an end-to-end manner [24]. It is largely composed of two parts, feature extraction and a clustering layer, which are jointly linked to optimize the resulting clusters. Feature extraction for dimensionality reduction curtails the high-dimensional kinematic information onto a latent space, which includes a meaningful representation [25]. This set of meaningful features goes to a temporal clustering layer with a specific similarity metric. In order to select an optimal number of clusters suitable for gait pattern analysis, a measure that evaluates clustering performance, such as silhouette score, is derived for each recursive process as the number of clusters changes. Figure 1 represents the overall architecture of DTCGP, which includes the DTC with gait cycle data.

#### 2.3.1. Feature Extraction

Unlike traditional ML pipelines, feature extraction from raw temporal input proceeds automatically via feature vectors on the latent space. This latent representation is achieved using Temporal AutoEncoder (TAE), which differs from existing encoders due to its intrinsic LSTM layers.

Before the main clustering, TAE was pretrained to initialize the centroids of each cluster for meaningful values. Initially, the input $x_{i}$, where *i* indicates the instance, moves to the encoder of TAE, which consists of a single one-dimensional (1D) convolution layer followed by two Bidirectional LSTMs (Bi-LSTMs). The 1D convolution layer with a kernel size of 10 and stride of 1 has a Leaky ReLU for the activation function and downsamples the data through max pooling with a selected pooling size. Subsequently, two Bi-LSTMs learn the temporal property of $x_{i}$ in both the forward and backward directions [24]. The resultant latent feature *z* is used for the main temporal clustering of the gait cycles. Throughout the decoder of TAE, which upsamples the latent features via the deconvolution layer, the encoded data are reconstructed as $\hat{x_{i}}$. Note that the upsampling layer has a same pooling size as the max pooling and a transposed convolution layer with a kernel size of 10, and a stride of 1 is used for deconvolution.To optimize TAE, the Mean Squared Error (MSE) value with the original input $x_{i}$ is computed, reconstructed as $\hat{x_{i}}$ with the total number of instances *N*, as follows:

(1) $$J_{MSE}=\frac{1}{N}\sum_{i=1}^{N}{\left(x_{i}-\hat{x_{i}}\right)}^{2}$$

#### 2.3.2. Clustering

Throughout the clustering layer, the latent features of the gait cycles extracted from TAE were used to update the centroids of the clusters. First, the centroids of each cluster are initialized using the results of a pre-trained TAE and agglomerative clustering with complete linkage. Subsequently, the similarity between the latent feature *z* and the initialized centroid *c* is calculated based on Pearson’s correlation $\rho_{z,c}$ as follows [26]:

(2) $$similarity\left(z,c\right)=\sqrt{2(1-\rho_{z,c})}$$

In this case, the formula for Pearson’s correlation is

(3) $$\rho_{z,c}=\frac{cov(z,c)}{\sigma_{z}\sigma_{c}}=\frac{E[\left(z-\mu_{z}\right)\left(c-\mu_{c}\right)]}{\sigma_{z}\sigma_{c}}$$

where cov denotes covariance, σ denotes the standard deviation, E denotes the expectation, and μ denotes the mean.

The similarity is then converted into a soft assignment probability $q_{ij}$ using Student’s t distribution [27], as shown below:

(4) $$q_{ij}=\frac{{\left(1+similarity\left(z_{i},c_{j}\right)\right)}^{-1}}{\sum_{j=1}^{K}{\left(1+similarity\left(z_{i},c_{j}\right)\right)}^{-1}}$$

Here, $q_{ij}$ represents the likelihood of assigning sample *i* to cluster *j* and *K* is the total number of clusters.

To enhance the clustering performance and emphasize high-confidence assignments, an auxiliary target distribution $p_{ij}$ that amplifies well-clustered points while reducing the impact of ambiguous data is defined by the square of $q_{ij}$ and $f_{j}$:

(5) $$p_{ij}=\frac{\frac{q_{ij}^{2}}{f_{j}}}{\sum_{j=1}^{K}\frac{q_{ij}^{2}}{f_{j}}}$$

where $f_{j}=\sum_{i=1}^{K}q_{ij}$ represents the cluster frequency. This target distribution is designed to focus the centroid update process on points that are more confidently assigned to clusters, thereby minimizing the influence of low-confidence assignments [28].

The clustering objective is formulated by minimizing the Kullback–Leibler (KL) divergence between the target distribution $P_{i}$ and the current soft assignment $Q_{i}$. Note that $P_{i}$ and $Q_{i}$ indicate the collections of the probability distributions $p_{i}$ and $q_{i}$, respectively.

(6) $$J_{KL}=\sum_{i=1}^{N}KL(P_{i}\left|\right|Q_{i})=\sum_{i=1}^{N}\sum_{j=1}^{K}p_{ij}\log\frac{p_{ij}}{q_{ij}}$$

Minimizing $J_{KL}$ encourages the cluster centroids to shift towards regions where data points with higher confidence are located, iteratively refining the cluster boundaries.

#### 2.3.3. Simultaneous Optimization

The two resulting losses from TAE and the clustering layer are both considered to train the DTC network. To optimize the weights for TAE, which eventually generates the latent features for clustering with Stochastic Gradient Descent (SGD), MSE loss and KL divergence loss are added. The total cost function $J_{total}$ obtained using Equations (1) and (6) is expressed as follows:

(7) $$J_{total}=J_{MSE}+J_{KL}$$

The two sequential steps of feature extraction and clustering affect each other, and it is clear that unsupervised clustering with time-series joint-level gait cycles is performed in a fully end-to-end manner.

## 3. Results

### 3.1. Hyperparameter Tuning

To achieve a certain level of clustering performance, the DTC model was fine-tuned during training. The resulting hyperparameters are listed in Table 2. The grid search method was used to select the corresponding values. The searching ranges for each hyperparameter are shown in square brackets, and the tuned values are shown in bold. The TAE was pre-trained over 10 epochs optimizing via Adam [29] before clustering. The overall training procedure was run for 600 epochs, which guaranteed convergence of the training loss. Because the TAE and temporal clustering layer are jointly optimized, two distinct learning rates for each optimizer are required. The pooling size determines the dimension of the latent features, and the hidden sizes of the two Bi-LSTMs at the encoder are fixed to 50 and 1, respectively, which are commonly selected parameters for DTC because it is difficult to cope with specific tuning in unsupervised learning [24].

### 3.2. Clustering of Gait Groups

To select the optimal number of clusters, the silhouette score (SS) is used, which is an internal validation criterion for the unknown underlying structure of the dataset [30]. By calculating the Euclidean distance between each data point and its surroundings, SS shows how well certain data are gathered within a cluster. Assuming that *i* is an individual sample point, silhouette score SS is represented as follows:

(8) $$SS=\frac{1}{N}\sum_{i=1}^{N}\frac{b(i)-a(i)}{max(a(i),b(i))}$$

where a(i) is the average distance between point *i* and other points in the same cluster, and b(i) is the minimum average distance between point *i* and other points outside the cluster. A score of 1 implies that the datum is in the proper cluster, and −1 indicates that it is not clustered well.

By varying the number of target clusters, the number of the optimal clusters was determined based on SS values. In general, the silhouette score tends to decrease as the number of clusters increases [31]. It is desirable to obtain fine-grained clusters to understand the different gait patterns of patients. Therefore, the case with the largest possible number of clusters and a high silhouette score is preferable.

**Figure 2:**
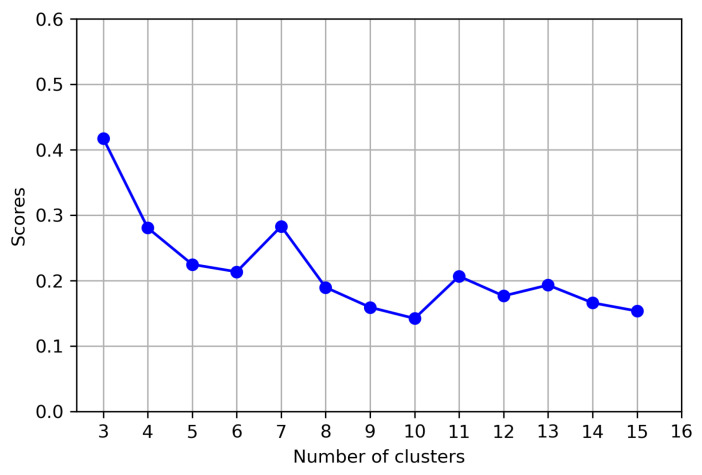
Silhouette score per number of clusters.

**Figure 3:**
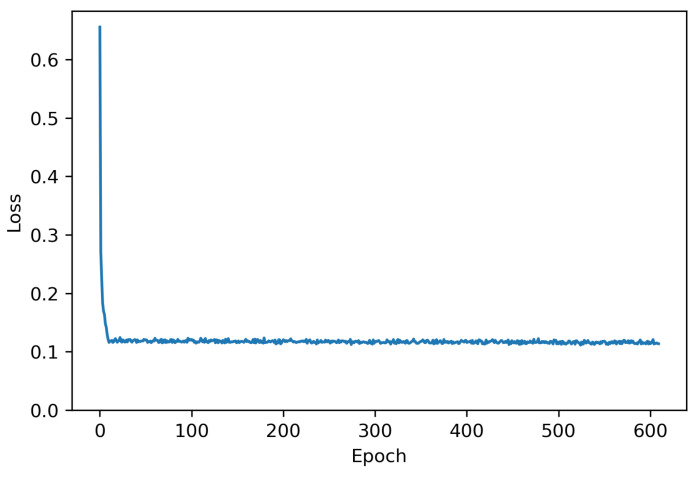
Loss curve for the optimal 7 clusters representing both pre-training and training.

Figure 2 shows the overall silhouette scores for the number of clusters between 3 and 15. The peak values are shown at 3, 7, 11, and 13. In order to improve clarity in the analysis, clusters with a single member were treated as outliers and excluded from the result and further analysis [32]. Thus, even after excluding the noise clusters, we aimed to maintain a high silhouette score while maximizing the number of clusters at the same time. As a result, after removing one cluster with a single instance from the number of clusters of seven with the silhouette score of 0.2831, which satisfies both conditions, we finalized six clusters as the optimal number of clusters for gait analysis. Figure 3 shows the loss curve at optimal clusters during 10 pre-training and 600 training epochs using the DTC model.

### 3.3. Characteristics of Selected Gait Groups

To understand the characteristics of the six selected groups, the statistics of gait features in spatiotemporal, kinematic, and clinical aspects are shown in Table 3. Note that SLS as a spatiotemporal feature refers to single limb support, and the total instances are 218 due to the dropout of the one cluster with a single member.

Specifically, among spatiotemporal features, Group D has the highest averaged velocity of 85.26 cm/s (±16.21) and Group B has the lowest one of 33.13 cm/s (±14.83). In the averaged cadence, Group D shows the highest value of 96.13 steps/m (±10.06) and Group B of 63.90 steps/m (±13.76) shows the lowest value. The remaining features can be observed in the table. Considering kinematic features, Group A has the highest averaged peak hip flexion of 47.08 deg (±9.30) and Group C has the lowest one of 28.99 deg (±9.17). In the averaged peak hip extension, Group B represents the highest value of 16.31 deg (±8.11) and Group C of −8.61 deg (±5.59) represents the lowest value. As clinical features, the motor function of post-stroke patients was evaluated using the motor sub-scale of the Fugl–Meyer Assessment (FMA) [33] and Functional Ambulation Categories (FAC) [34]. The averaged FMA score is the highest for Group F of 86.33 (±16.16), and Group B’s score of 57.12 (±24.05) is the lowest. In the averaged FAC score, Group D shows the highest value of 4.56 (±0.67) and Group B shows the lowest value of 3.38 (±1.19). Note that the characteristics of the remaining clusters can be interpreted differently from the desired perspective.

To determine if the selected gait groups were clinically significant, an analysis of variance (ANOVA) test was performed with the gait features in Table 3. Using Python’s statsmodels [35], one-way ANOVA was applied to spatiotemporal, kinematic, and clinical features. Table 4 represents the result of an ANOVA test which includes F-values and *p*-values for all gait features. The *p*-values for all tests are less than 0.05, demonstrating that the six selected gait groups are statistically significant and clinically meaningful [36].

The gait abilities of the selected groups were evaluated by comparing them to a control group. The control group data were sourced from the default dataset provided by the Orthotrak motion capture system [18], representing the average joint trajectory values for healthy European adults. Despite the use of European reference data, the kinematic similarities between Korean and European populations render this comparison valid for gait analysis [37]. No control data were used during the clustering or model training phases. This control dataset was used exclusively for post hoc analysis to assess the clustering results. Specifically, the control data were applied to visualize gait differences and compute the Root Mean Square Error (RMSE) of joint angle trajectories, providing a quantitative measure of deviation from normative gait patterns. The RMSE score is calculated using Equation (9), where *N* is the total number of gait cycle points across hip, knee, and ankle joints, $y_{i}$ is the joint angle from the patient, and $\hat{y_{i}}$ is the joint angle from the control group.

(9) $$\mathrm{RMSE}=\sqrt{\frac{\sum_{i=1}^{N}{\left(y_{i}-\hat{y_{i}}\right)}^{2}}{N}}$$

Therefore, if a particular group follows the normal gait trajectory, the RMSE value is low; if not, the RMSE value is high.

**Figure 4:**
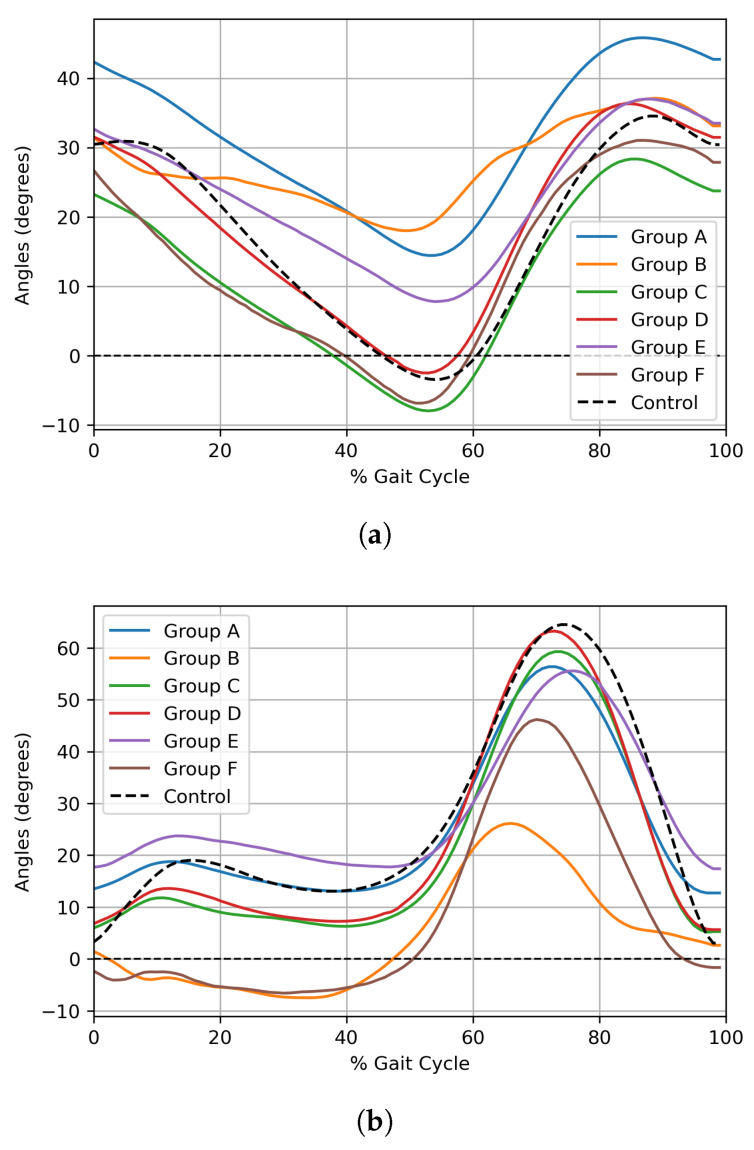

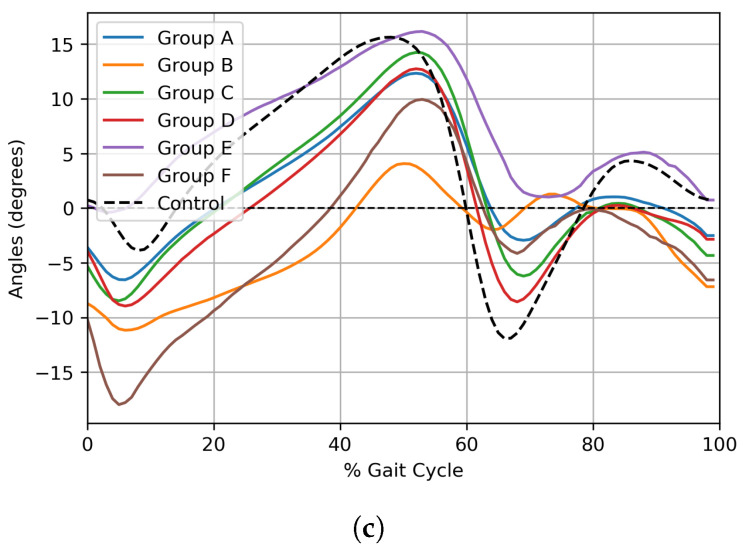
Average joint-level angular trajectories for each cluster on the affected side. (**a**) Hip, (**b**) Knee, (**c**) Ankle.

Figure 4 depicts the average joint angle trajectories on the affected side of the hip, knee, and ankle for each cluster, alongside the corresponding trajectories from the control group, and Table 5 lists the RMSE scores for the six groups. The control group is marked with a dotted line in order to be distinguished from the optimal clusters, and only the group averages are displayed as curves without their standard deviations to enhance visibility. In examining the gait patterns at hip, knee, and ankle joints across the six groups, distinct differences emerge in relation to the control group’s trajectories. Group A shows a trajectory at the knee joint closely aligned with the control but exhibits reduced peak dorsiflexion in the ankle and generally higher hip joint angles. Group B has the largest RMSE value of 17.6484, indicating the greatest deviation from the control group, with minimal differences in peak flexion and extension across the hip, knee, and ankle, resulting in the worst walking ability among six groups. Group C presents hip and knee joint trajectories that resemble the control, though the ankle shows reduced peak dorsiflexion. Group D displays the smallest RMSE value at 4.7918, indicating that its joint angle trajectories across all joints were closest to the control. Group E features a relatively large peak extension in the hip joint and reduced peak dorsiflexion in the ankle. Lastly, Group F lacks a loading response in the knee during the stance phase and shows reduced peak dorsiflexion in the ankle.

### 3.4. Comparison of Joint Angle Trajectories on Affected and Unaffected Sides

**Figure 5:**
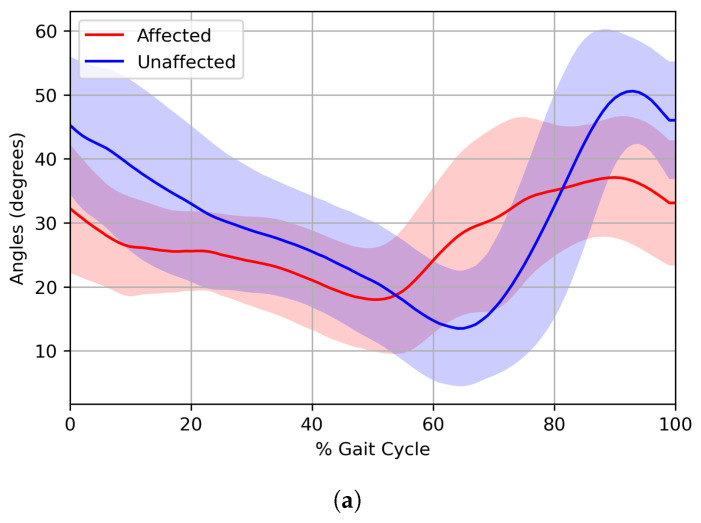

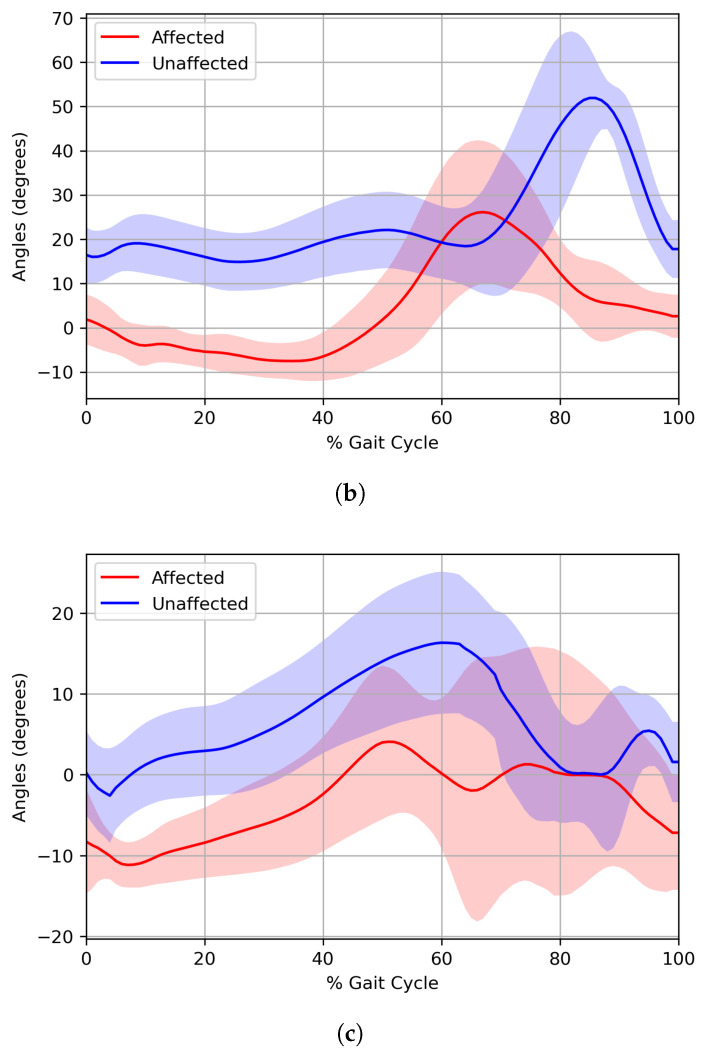
Averaged joint angle trajectories for Group B on both affected and unaffected sides. (**a**) Hip, (**b**) Knee, (**c**) Ankle.

The averaged joint angle trajectories for Group B with the worst gait ability on the affected and unaffected sides are presented in Figure 5. In the figure, the trajectories for affected and unaffected sides are shown with different colors as solid lines and the standard deviation (1SD) as a shaded area. In the trajectories of the hip, the peak hip flexion of the unaffected side had a much larger angle value than the affected side. On the other hand, the peak hip extension was smaller on the unaffected than the affected side. This indicates that the arc containing extension and flexion was collapsed on the affected side of the hip. In addition, the starting angle of the hip joint was higher than that of the affected side and followed the angle of the normal control group. For knee kinematics, peak knee extension was not observed clearly at the stance phase of the affected; therefore, the loading response was poorly performed. In addition, in the swing phase, the angle of peak knee flexion from the unaffected side was larger than that of the affected side. At the ankle joint, the overall angle values of the unaffected were much larger than those of the affected side. Specifically, in the case of the affected side, the arc was collapsed; thus, the values of dorsiflexion and plantar flexion became very small compared with those of the unaffected side. Furthermore, on the affected side, dorsiflexion was poorly formed as the affected ankle joint could not reach the peak. Group B with the worst walking ability shows severe gait asymmetry between the affected and unaffected side.

## 4. Discussion

To illustrate the performance of DTCGP, Table 6 compares the silhouette scores of DTCGP to those of other machine learning-based methods, i.e, *k*-Means clustering, *k*-Shape [38], and simultaneous clustering and classification (SCC) [8]. Each row represented the silhouette scores at a different number of clusters. The *k*-Means clustering and *k*-Shape methods were implemented using tslearn [39] and the same trajectory data in this study. For the *k*-Means clustering method, three types of distance metrics were used to derive specific features from time-series data. Not only the clustering with the Euclidean (eucli.) distance [40], but also with dynamic time warping (dtw), which extracts the similarity by finding the optimal alignment between each sample, allowing stretching or compressing of the time axis [41], were considered. Moreover, clustering with soft dtw (softdtw), which enhances the dtw approach by introducing a differentiable variant of the DTW distance measure [42], was also used for comparison. The results of SCC were directly referenced from [8]. The DTCGP model’s performance benefits from the simultaneous optimization of both the reconstruction accuracy of the autoencoder and the cluster quality based on probabilistic similarity [43]. As a result, the DTCGP outperformed the others in most cases and achieved the highest silhouette values, except in a few cases.

While the silhouette score of 0.2831 from DTCGP, representing the value for the selected optimal cluster, may appear modest, it is important to note that SS is a relative clustering validity index [44,45]. The selection of this cluster reflects the relative quality of clustering in this study, highlighting that even moderate SS values can provide meaningful insights when balancing high SS with maximizing the number of clusters. Furthermore, when comparing SS across various clustering algorithms, the selected model consistently outperformed alternative methods, reinforcing the validity of the clustering results.

Among several studies using DL on rehabilitation, [2] has the same purpose as DTCGP, which is to establish a personalized treatment strategy for post-stroke patients based on their gait patterns. They utilized a model with multiple layers for computational benefits, which consisted of a detection part for judging abnormal gaits and a classification part for dividing the gaits of eight stroke patients into four specific types of impairment. Compared to the case of simply using a fully connected layer, DTCGP has convolutional and Bi-LSTM layers, which have advantages in extracting latent features and interpreting the temporal relationship between these features [24]. Also, they classify stroke gait patterns into four fixed labels, which are drop foot, circulation, hip hiking, and back knee. On the other hand, DTCGP can change the number of clusters based on performance evaluation, allowing the pattern to be analyzed more flexibly in various situations.

Kim et al. [8] applied simultaneous clustering and classification (SCC) to identify gait patterns in post-stroke hemiplegic patients using kinematic data for a gait cycle. While both approaches, DTCGP and SCC, effectively clustered gait data, the key distinction lies in the preprocessing stage. SCC involves manual intervention through the extraction of kinematic features, while DTCGP applies end-to-end deep learning without human involvement, enhancing accuracy, consistency, and reducing potential bias [10,11,13]. Moreover, the DTCGP model consistently achieved higher silhouette scores compared to SCC, as presented in Table 6, indicating improved cluster separation and overall performance. This trend highlights the model’s ability to capture diverse gait patterns, potentially identifying asymmetrical gait characteristics that may be less distinguishable through traditional methods like SCC.

Furthermore, the proposed method using DTC has the potential to significantly impact clinical practice. The method technically demonstrates the significance in leveraging unsupervised learning to identify clinically meaningful clusters, especially in medical analysis where clinical ground truth is often unavailable. This approach can be applied to monitoring and diagnosing irregular patterns by directly utilizing raw time-series data, such as detecting anomalies in arrhythmias using ECG time series data [46,47]. Also, the gait characteristics of the selected groups derived from the proposed method can be applied to personalized treatments, aligning with each patient’s unique recovery tendency.

This study has limitations that should be addressed in the next phase. To fully reflect the complex characteristics of walking, it is necessary to analyze data from all anatomical planes. Since only joint angles and angular velocity trajectories in the sagittal plane were used in this study, incorporating data in the coronal and transverse planes would improve the clustering. Additionally, the use of muscle activity and gait symmetry will allow for more accurate gait analysis. Because cross-sectional studies cannot explain the causal relationship between motor function and clinical duration [48], further research is needed that considers the long-term perspective of motor function recovery.

Future studies could analyze a broader range of gait patterns by including gait data from chronic patients or healthy individuals in addition to sub-acute patient data. Additionally, DL-based techniques can be used to study longitudinal recovery patterns over a longer period of time, given the six months of clinical data available for each patient. Furthermore, the longitudinal recovery pattern analysis results can aid in developing personalized treatment strategies that complement various rehabilitation treatment methods.

## 5. Conclusions

This study presents a deep learning approach for clustering gait patterns of post-stroke hemiplegic patients from a cross-sectional perspective. The main contributions of this study are clarified as follows. First, the complex joint angle and angular velocity trajectory data of hemiplegic post-stroke patient gaits were directly processed using the deep temporal clustering method in an end-to-end manner minimizing human intervention. This approach not only improves accuracy, but also improves efficiency by reducing the amount of work required to analyze complex gait patterns. Second, in this study, we presented a DTC model optimized for clustering gait patterns of post-stroke hemiplegic patients. Third, the selected gait groups were confirmed to be clinically significant by an ANOVA test with clinical factors in patients’ gaits.

## Figures and Tables

**Table 1:** The number of instances of kinematic data after onset.

Weeks	Patients	Instances
2	4	4
3	5	10
4	4	12
6	5	20
8	9	45
10	5	30
12	6	42
24	7	56
**Total**		219

**Table 2:** Hyperparameters for grid search.

Hyperparameter	Value
epochs (Pre-train)	10
epochs	600
batch size	[32, 64, 128]
learning rate (TAE)	[0.01, **0.001**, 0.0001]
learning rate (Cluster)	[$10^{-4}$, $10^{-5}$, $10^{-6}$]
pooling size	[8, **10**, 12]
hidden size (Bi-LSTM 1)	50
hidden size (Bi-LSTM 2)	1

The optimal values are highlighted in **bold**.

**Table 3:** Spatiotemporal, kinematic, and clinical characteristics of optimal gait groups. Aff. and unaff. refer to affected and unaffected side, respectively.

Features	Unit	Total	Group A	Group B	Group C	Group D	Group E	Group F
		**(218)**	**(113)**	**(8)**	**(26)**	**(50)**	**(15)**	**(6)**
**Spatiotemporal**								
Velocity	cm/s	69.03 ± 25.48	65.97 ± 25.34	33.13 ± 14.83	70.37 ± 24.68	85.26 ± 16.21	56.55 ± 27.93	64.69 ± 16.25
Cadence	steps/m	89.86 ± 17.54	90.61 ± 17.46	63.90 ± 13.76	87.52 ± 19.59	96.13 ± 10.06	86.87 ± 22.69	75.62 ± 11.47
Stride length	cm	90.30 ± 23.68	85.40 ± 23.50	61.11 ± 18.38	95.50 ± 20.13	106.12 ± 15.08	76.22 ± 25.53	102.25 ± 9.98
Step length (aff.)	cm	46.06 ± 11.46	43.92 ± 11.10	37.00 ± 9.29	47.36 ± 11.38	53.48 ± 7.20	37.43 ± 14.18	52.55 ± 5.99
SLS (aff.)	%	31.52 ± 6.52	31.09 ± 6.77	20.58 ± 5.07	32.39 ± 4.53	34.64 ± 4.15	29.91 ± 8.11	28.49 ± 3.21
Stance (aff.)	%	65.29 ± 4.43	65.75 ± 4.52	60.51 ± 4.91	65.43 ± 4.09	64.11 ± 2.58	68.69 ± 6.13	63.82 ± 4.05
Step length (unaff.)	cm	44.15 ± 13.36	41.27 ± 13.56	24.88 ± 11.87	47.97 ± 9.46	52.59 ± 8.68	38.83 ± 13.80	50.28 ± 3.65
SLS (unaff.)	%	34.71 ± 4.43	34.25 ± 4.52	39.49 ± 4.91	34.57 ± 4.09	35.89 ± 2.58	31.31 ± 6.13	36.18 ± 4.05
Stance (unaff.)	%	68.48 ± 6.52	68.91 ± 6.77	79.42 ± 5.07	67.61 ± 4.53	65.36 ± 4.15	70.09 ± 8.11	71.51 ± 3.21
**Kinematic**								
Peak hip flexion	deg	41.20 ± 10.90	47.08 ± 9.30	39.41 ± 10.25	28.99 ± 9.17	36.65 ± 6.53	37.39 ± 10.38	33.26 ± 6.91
Peak hip extension	deg	6.12 ± 12.13	13.44 ± 10.22	16.31 ± 8.11	−8.61 ± 5.59	−2.88 ± 4.37	6.33 ± 4.85	−7.18 ± 3.97
Peak knee flexion (stance)	deg	17.08 ± 8.67	20.41 ± 6.53	−1.39 ± 4.63	12.30 ± 6.65	14.81 ± 6.39	24.70 ± 6.40	−0.52 ± 5.21
Peak knee extension (stance)	deg	8.69 ± 8.87	12.01 ± 7.85	−8.37 ± 4.55	4.28 ± 5.97	5.83 ± 5.94	16.43 ± 4.77	−7.54 ± 4.61
Peak knee flexion (swing)	deg	58.93 ± 13.13	59.04 ± 12.54	28.85 ± 14.46	60.54 ± 8.85	64.15 ± 7.49	58.19 ± 15.19	48.28 ± 14.50
Peak knee extension (swing)	deg	8.94 ± 8.50	11.62 ± 6.68	−0.58 ± 6.05	4.30 ± 6.30	5.02 ± 5.80	19.49 ± 12.39	−2.36 ± 8.22
Peak plantar flexion	deg	20.12 ± 7.71	18.73 ± 7.38	13.97 ± 15.56	23.18 ± 5.64	23.03 ± 5.96	19.98 ± 8.58	17.27 ± 7.79
Peak dorsiflexion	deg	8.67 ± 5.16	7.66 ± 5.04	8.30 ± 6.25	9.31 ± 3.72	10.90 ± 5.58	8.50 ± 4.96	7.32 ± 3.45
**Clinical**								
FMA		79.50 ± 19.32	78.02 ± 21.36	57.12 ± 24.05	80.85 ± 16.34	85.98 ± 11.62	75.87 ± 17.66	86.33 ± 16.16
FAC		4.09 ± 1.00	4.01 ± 1.02	3.38 ± 1.19	4.15 ± 0.83	4.56 ± 0.67	3.40 ± 1.18	4.17 ± 1.33

**Table 4:** ANOVA results for all gait features.

	ANOVA Result	
	**F-Value**	***p*** **-Value**
Velocity	10.0742	<0.0001
Cadence	6.5343	<0.0001
Stride length	11.8456	<0.0001
Step length (affected)	9.7703	<0.0001
SLS (affected)	8.7701	<0.0001
Stance (affected)	5.1544	0.0002
Step length (unaffected)	11.9162	<0.0001
SLS (unaffected)	5.1544	0.0002
Stance (unaffected)	8.7699	<0.0001
Peak hip flexion	24.4284	<0.0001
Peak hip extension	52.3391	<0.0001
Peak knee flexion (stance)	36.6886	<0.0001
Peak knee extension (stance)	29.2463	<0.0001
Peak knee flexion (swing)	14.1336	<0.0001
Peak knee extension (swing)	21.6299	<0.0001
Peak plantar flexion	4.5054	0.0006
Peak dorsiflexion	3.0462	0.0112
FMA	3.9357	0.0019
FAC	5.0359	0.0002

**Table 5:** Gait performance of each group compared to the control group.

Group	RMSE
A	9.3454
B	17.6484
C	6.3147
D	4.7918
E	6.6170
F	13.3559

**Table 6:** Comparison of clustering performance between DTCGP and other methods.

	No. of Clusters												
	**3**	**4**	**5**	**6**	**7**	**8**	**9**	**10**	**11**	**12**	**13**	**14**	**15**
*k*-Means (eucli.)	0.1913	0.1577	0.1287	0.1395	0.1263	0.1136	0.1520	0.1246	0.1303	0.1301	0.1249	0.1109	0.1194
*k*-Means (dtw)	0.1062	0.1566	0.1400	0.1012	0.0990	0.0982	0.0854	0.0627	0.0420	0.0382	0.0449	0.0377	0.0312
*k*-Means (softdtw)	0.2121	0.1530	0.1277	0.1218	0.1331	0.0970	0.0879	0.0939	0.0942	0.0769	0.0760	0.0887	0.0941
*k*-Shape	0.1950	0.1449	0.1533	0.1368	0.1252	0.1050	0.1127	0.1083	0.1126	0.0996	0.0981	0.0923	0.0857
SCC	0.2235	0.1517	0.1604	0.1765	0.1426	0.1312	0.1447	**0.1710**	0.1371	0.1443	0.1563	**0.1715**	**0.1572**
DTCGP	**0.4174**	**0.2806**	**0.2249**	**0.2136**	**0.2831**	**0.1898**	**0.1593**	0.1423	**0.2068**	**0.1767**	**0.1933**	0.1662	0.1536

The largest silhouette scores per number of clusters are highlighted in **bold**.

## Data Availability

All datasets have been anonymized to protect patient confidentiality. The data are not publicly available due to the regulation of SMC Institutional Review Board (SMC 2017-11-081).
